# Precise identification of Dirac-like point through a finite photonic crystal square matrix

**DOI:** 10.1038/srep36712

**Published:** 2016-11-18

**Authors:** Guoyan Dong, Ji Zhou, Xiulun Yang, Xiangfeng Meng

**Affiliations:** 1 College of Materials Science and Opto-Electronic Techology, University of Chinese Academy of Sciences, Beijing 100049, P. R. China; 2 State Key Laboratory of New Ceramics and Fine Processing, School of Materials Science and Engineering, Tsinghua University, Beijing 100084, China; 3 Department of Optics, Shandong University, Jinan, 250100, China

## Abstract

The phenomena of the minimum transmittance spectrum or the maximum reflection spectrum located around the Dirac frequency have been observed to demonstrate the 1/L scaling law near the Dirac-like point through the finite ribbon structure. However, so far there is no effective way to identify the Dirac-like point accurately. In this work we provide an effective measurement method to identify the Dirac-like point accurately through a finite photonic crystal square matrix. Based on the Dirac-like dispersion achieved by the accidental degeneracy at the centre of the Brillouin zone of dielectric photonic crystal, both the simulated and experimental results demonstrate that the transmittance spectra through a finite photonic crystal square matrix not only provide the clear evidence for the existence of Dirac-like point but also can be used to identify the precise location of Dirac-like point by the characteristics of sharp cusps embedded in the extremum spectra surrounding the conical singularity.

During the last few years the Dirac cone dispersions in various periodic systems have attracted significant attention for many remarkable wave transport properties^1^,^2^,^3^,^4^. Particularly important is the discovery of graphene with the two-dimensional (2D) honeycomb structures^5^,^6^,^7^, in which the conduction band and the valence band touch each other as Dirac cones at the Dirac point (DP), leading to some intriguing electronic transport properties. By proper design and fabrication, photonic crystals (PhCs) can also exhibit Dirac cones at the corners of the Brillouin zone of the triangular and honeycomb lattices^8^,^9^,^10^,^11^,^12^,^13^,^14^, leading to many unusual transmission properties, such as the classical analogs of Zitterbewegung^15^, pseudodiffusion^8^,^16^ and extinction of coherent backscattering^17^. Since the accidental degeneracy of two dipolar modes and a single monopole mode generates at the Dirac-like point (DLP), the linear dispersions of Dirac cone can also occur at the Brillouin zone center of PhCs^18^,^19^,^20^,^21^,^22^,^23^. The PhC can mimic the zero-index medium (ZIM) with the characteristics of uniform field distribution, which can be understood from the effective medium perspective^18^. Compared with the DP induced by double degeneracy near the corner points of the triangular/honeycomb lattice, linear dispersions near the center point induced by the triple degeneracy display many unique scattering properties, such as conical diffraction^21^,^22^,^23^, wave shaping and cloaking^18^,^19^,^20^. Different from the conventional metamaterials^24^,^25^ comprising metallic components whose high inherent losses may actually reduce the functionality of any proposed device, PhCs can be made entirely of dielectric or semiconductor materials which have the evident benefits in terms of low loss.

The PhC ribbon with two parallel edges has been studied near the DP due to its intriguing transport properties^9^,^16^,^26^. The dispersion properties obeying the 1/L scaling law near the DP or DLP in the normal propagation direction have been verified theoretically and experimentally^21^,^26^,^27^ through the dielectric PhC ribbons with the finite thicknesses, which come from the conically shaped dispersion and the transmission as a function of frequency with an extremum near the conical singularity^8^,^26^. Although the transmission properties can be used to demonstrate the existence of Dirac cone, it is difficult to distinguish the precise crossing point from the wide extremum range just relying on the transmittance spectrum. Here we proposed an effective method to identify the conical singularity of the DLP accurately by the measurement of transmittance spectra through a finite PhC square matrix embedded in free space with four open boundaries. The numerical simulation and microwave experiment verified that the nontrivial wave transport property with the sharp cusps embedded in the extremum spectra can be achieved through the finite PhC square matrix to provide a clear evidence for the existence of DLP and indicate the precise location of DLP specifically.

## Results

### Design and numerical simulations

**Figure 1:**
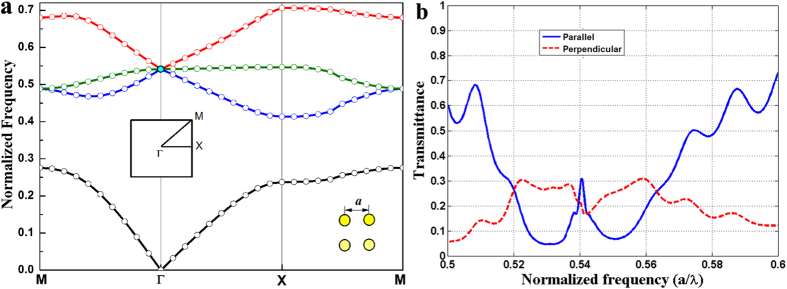
Band diagram and simulated transmittance spectra through the 10*a* × 10*a* PhC square matrix. (**a**) Band structure of TM waves for the 2D square-lattice PhC with a crossing point at the Brillouin zone center indicated by the cyan point at the normalized frequency *ω*_D_ = 0.541. (**b**) The simulated transmission spectra in the directions parallel (indicated by blue solid lines) and perpendicular (indicated by red dash lines) to the incident propagation direction.

The sample of square-lattice PhC was composed of dielectric rods embedded in air with the lattice constant *a* = 1, cylinder radius *r* = 0.2*a*, relative permittivity *ε*_*r*_ = 12.5 and permeability *μ* = 1. The corresponding band diagram of the infinite PhC for the transverse magnetic (TM) polarization with the electric field *E*(*x, y*)*e*^*iωt*^ along the rod axis is shown in Fig. 1a, where the frequency is a normalized quantity *a*/λ (“*a*” is the lattice constant, “λ” is the wavelength of incident wave). TM2 and TM4 bands cross each other linearly at the Brillouin zone center Γ to form a Dirac cone intersected by the additional flat TM3 band at the degenerate point, i.e. DLP (indicated by the cyan point). As mentioned in the introduction, the Dirac cone is created at the center of the Brillouin zone with *k* = 0 and the effective zero-refractive-index (*n*_*eff*_) with *ε*_*eff*_ = *μ*_*eff*_ = 0 can be achieved at the Dirac frequency of *ω*_D_ = 0.541.

Numerical simulations have been introduced to investigate the transmittance spectra through the PhC square matrixes. A Gaussian pulse (the frequency span 0.5~0.6 *a*/λ) of TM mode with the waist width of 5*a* was placed in front of the 10*a* × 10*a* PhC square matrix with the beam normally incident upon the input interface, thus three emergent beams can be measured out of the other three output interface of the PhC square matrix, where one parallel transmittance spectrum and two symmetrical upward and downward (i.e. perpendicular) transmittance spectra were obtained by the ratio between the transmitted power of the output interface and the incident power. The average power flow was computed by spatially integrating the energy flux S(*ω*), i.e. the Poynting vector. The average power flux is defined by the following formula, 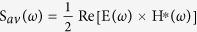. As shown in Fig. 1b, the parallel transmittance spectrum (indicated by blue solid line) opened a wide stopband around the Dirac frequency of *ω*_D_ = 0.541 in the similar form of the transmission anti-resonance^8^,^22^ near the DLP through the PhC ribbons. While the primary difference was a sharp cusp appeared at the frequency of *ω* = 0.54 in the bottom of the parallel transmittance spectrum with a little bit deviation from the Dirac frequency *ω*_D_ for the finite-size effect. In the perpendicular direction, the upward and downward energy leakages of photon have been measured with the similar transmission spectrum due to the symmetry of this measurement system. Therefore, only the unilateral perpendicular transmittance spectrum (indicated by the red dash line) was shown in Fig. 1b with a concave cusp at the Dirac frequency *ω*_D_ = 0.541.

### Experimental results

**Figure 2:**
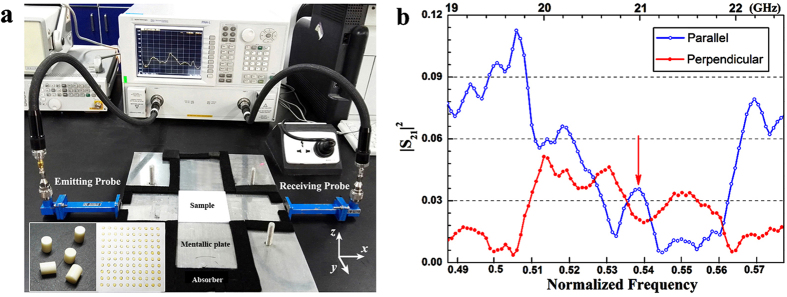
Experimental layout characterization and experimental results. (**a**) Internal structure photograph of experimental layout with a microwave measurement system. (**b**) The experimental measured transmittance spectra.

To further verify the above mentioned simulation results, we measured the transmission properties of electromagnetic wave (EMW) through a PhC square matrix experimentally. The sample of the square-lattice PhC square matrix with the size of 10*a* × 10*a* was composed of 100 highly pure Al_2_O_3_ ceramic cylinders with the diameter *d* = 3.3 mm, height *h* = 4.3 mm, permittivity *ε*_r_~10, permeability *μ*_r_ = 1 and dielectric loss tangent tan δ~10^−5^, which were embedded in the background ABS square matrix with the lattice constant *a* = 7.7 mm. Figure 2a gives the internal structure photograph of the experimental layout with a microwave measurement system, where the upper and lower metallic plates form a planar waveguide to ensure the TEM mode invariable between the plates along the *z* axis. The sample of PhC matrix was placed in the middle of the cross-shape light pathway surrounding by the absorbing materials to guide EMWs travelling in straight lines. Two waveguide adapters with the cross-sectional dimension of 10.7 mm × 4.3 mm were utilized as the emitting and receiving antennas to ensure only the dominant mode of TE_10_ in *K*-band (18~26 GHz) propagates in the waveguides, which were connected to a vector network analyzer (VNA, Agilent N5230C) to measure the transmittance (|S_21_|^2^) spectra.

Figure 2b shows the measured transmittance spectra with the parallel transmittance indicated by the blue hollow dot line and the perpendicular unilateral transmittance indicated by the red solid dot line. It is clear that the transmittance spectrum parallel to the incident direction presents low transmittance in the frequency range of 19.9~22 GHz (i.e. normalized frequency 0.51~0.565) with an obvious peak emerging from the minimum transmittance spectrum at 21 GHz (i.e. normalized frequency 0.539*a*/λ). On the contrary, the perpendicular transmittance spectrum presents high transmittance in the similar frequency scope with a dip cusp embedded in the flat peak at 21.04 GHz (i.e. normalized frequency 0.54 *a*/λ). These experimental results are in reasonable agreement with the simulation results, though the transmittances are lower than the expected due to the size mismatch of different waveguides and there exist some small split peaks due to the fabrication imperfections.

### Comprehensive analysis

**Figure 3:**
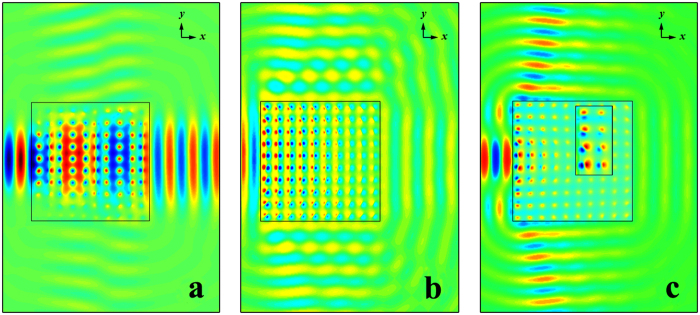
Evolution of field distribution at different representative frequencies. Evolution of field distribution for the Gaussian beam through the 10*a* × 10*a* PhC square matrix at three representative frequencies of (**a**) *ω* = 0.5, (**b**) *ω* = 0.54 and (**c**) *ω* = 0.55 with the normalized frequency unit of *a*/λ.

Although the sharp cusps have emerged in the extremum spectra through the PhC square matrix, we are still not sure the sharp cusps can indicate the DLP exactly. The spatial evolution of the field distribution at the different representative frequencies was investigated in the aforementioned measurement system and shown in Fig. 3a–c. Within the passband of the parallel transmittance spectrum, such as at the frequency of *ω* = 0.5, the propagation effect along the incident *x* direction can be seen clearly in Fig. 3a with little leakage in the *y* direction. When the frequency of incident beam was chose to be *ω* = 0.54 closing to the sharp cusps, as shown in Fig. 3b, the diffusive radiation field with uniform phase was excited in the PhC matrix and the outfield wavefronts were reshaped into the boundary shape of the PhC square matrix just like the ZIM, even though the field intensity distribution was not uniform due to the influence of edge states near the boundaries of the PhC matrix. Within the stopband of the parallel transmittance spectrum, such as at the frequency of *ω* = 0.55, the field intensity decaying exponentially along the incident propagation direction with two symmetrical leaky beams shooting out from the upper and lower boundaries to free space obviously in Fig. 3c.

For the incident light polarized along the *z* direction in free space, the Maxwell’s equation can be reduced to the Helmholtz equation


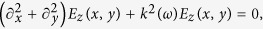


where the wave vector *k*(*ω*) can be extended as *k*(*ω*) = *k*(*ω*_D_) + (*ω* − *ω*_D_)/υ_D_ + β(*ω* − *ω*_D_)^2^ + … at *ω*_D_ > 0. For *k*(*ω*_D_) = 0 at the center of the Brillouin zone, neglecting the higher-order terms, we would have a linear dispersion *k*(*ω*) = (*ω* − *ω*_D_)/υ_D_ which is the necessary condition for a Dirac cone. When the frequency *ω* is close to the Dirac frequency *ω*_D_, owing to *k*^2^ = *k*_*x*_^2^ + *k*_*y*_^2^ → 0, the field distribution inside the PhC square matrix tends to be diffusive radiation. Within the passband of the parallel transmittance spectrum, *k*_*y*_ becomes imaginary for real *k*_*x*_ and the incident wave can pass through the PhC matrix perfectly with little leaky mode in the *y* direction; within the stopband of the parallel transmittance spectrum, the fields along the *x* direction decay exponentially from the incident interface, *k*_*y*_ becomes real for imaginary *k*_*x*_, i.e. *E*(*x, y*) = *E*_0_(*x, y*)*e*^−|*kx*|*x*^*e*^*i ky*·*y*^. Und*e*r the influence of the periodic boundary scattering which provide the additional momentum, the strong field localization near the incident interface may excite the lateral leaky modes to induce the leaky radiation from the upper and lower boundaries to free space. Since *ε*_eff_ and *μ*_eff_ approach zero simultaneously at the DLP, the PhC matrix can be regarded as an effective ZIM to become more transparent in the *x* direction at the conical singularity than the adjacent frequencies.

## Discussion

**Figure 4:**
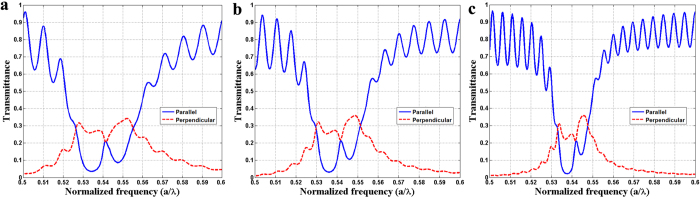
Influence of PhC matrix size on the transmittance spectra. Evolution of transmission spectra parallel (indicated by blue solid lines) and perpendicular (indicated by red dash lines) to the incident direction through the PhC square matrixes with the different sizes of (**a**) 15*a* × 15*a,* (**b**) 20*a* × 20*a*, (**c**) 30*a* × 30*a*.

The transmittance spectra were much affected by the bulk states and the leaky edge states of the finite PhC square matrix, which should have a close relation with the size of the PhC matrix; therefore the transmittance spectra through the PhC square matrixes with three different sizes of 15*a* × 15*a*, 20*a* × 20*a*, and 30*a* × 30*a* were measured respectively. In order to ensure the normal incident beam can be poured on the input interface of the PhC matrix completely and the leaky modes can be excited by the periodic boundaries, the incident Gaussian light source was placed in front of the PhC square matrix with a waist width 5*a* less than the edge length of the incident interface. As shown in Fig. 4a–c, no matter in the parallel or the perpendicular direction, the extremum (minimum and maximum) spectra nearby the DLP shrank gradually with the increasing of PhC matrix size. As long as the size is large enough, the PhC square matrix can be regarded as an infinite PhC structure with the same transmittance at different exit boundaries, which leads to the upward and downward sharp cusps embedded in the corresponding parallel and perpendicular extremum transmittance spectra intersecting at the frequency of DLP; thus the DLP can be identified accurately by the sharp cusps due to the property of uniform field distribution at the conical singularity.

In conclusion, in this work we provided an effective measurement system composed of a PhC square matrix and a normal incident Gaussian light source to identify the DLP accurately. Since the conical dispersion shape near the DLP brought about the exponential decaying fields in the incident propagation direction and the periodicity of the finite PhC matrix excited the lateral leaky radiation, the nontrivial transmission properties near the DLP induced the characteristics of sharp cusp embedded in the extremum transmittance spectra through the PhC square matrix. Both the simulated and experimental results demonstrate the measurement method of transmittance through the finite PhC square matrix makes it easy to identify the precise position of DLP from the wide extremum spectra around the conical singularity.

## Methods

The plane-wave expansion method was used to study the photonic band structure of the square-lattice PhC and the finite-difference time-domain (FDTD) method was used to calculate and simulate the transmission properties of the guided mode and leaky mode theoretically.

## Additional Information

**How to cite this article**: Dong, G.Y. *et al*. Precise identification of Dirac-like point through a finite photonic crystal square matrix. *Sci. Rep.* **6**, 36712; doi: 10.1038/srep36712 (2016).

**Publisher’s note:** Springer Nature remains neutral with regard to jurisdictional claims in published maps and institutional affiliations.
